# Clinical association of progesterone receptor isoform A with breast cancer metastasis consistent with its unique mechanistic role in preclinical models

**DOI:** 10.1186/s12885-020-07002-0

**Published:** 2020-06-03

**Authors:** Rayna Rosati, Kailey Oppat, Yanfang Huang, Seongho Kim, Manohar Ratnam

**Affiliations:** Barbara Ann Karmanos Cancer Institute and Department of Oncology, Wayne State University School of Medicine, 4100 John R, HWCRC 840.1, Detroit, MI 48201-2013 USA

**Keywords:** Breast cancer, Progesterone receptor, Estrogen receptor, Estrogen, Progesterone, Mifepristone

## Abstract

**Background:**

Luminal breast cancer (L-BCa) comprises the majority of incurable, distally metastatic breast cancer cases. Estrogen supports growth of L-BCa cells but suppresses invasiveness. Estrogen also induces the progesterone receptor (PR). Invasiveness and metastasis of L-BCa cells is supported by the short PR isoform (PR-A), in response to the range of pre- and post-menopausal plasma hormone levels, by counteracting the effects of estrogen via micro RNA-mediated cross-talk with the estrogen receptor (ER). PR-B directly supports L-BCa invasion and metastasis and also inhibits tumor growth, both only at high progesterone levels. As public datasets on L-BCa tumors cannot distinguish PR-A, this study was designed to seek clinical evidence for the role of PR-A in metastasis in comparison with PR-B and ER.

**Methods:**

Measurement of tumor PR-A, PR-B and ER mRNA expression in 125 treatment-naive primary L-BCa patients with differential node involvement and analysis using linear mixed effects models. Transcriptional activity assays of PR-A and PR-B.

**Results:**

Lymph node involvement was strongly associated with PR-A expression (median, 3-fold higher vs. node-negative), independent of age, pathologic type, tumor grade, HER2 and PR-B. PR-B and ER correlated weakly with PR-A, but whereas PR-B and the PR-A/PR-B ratio were not significantly associated with node involvement, ER weakly negatively correlated with node positivity. PR-A was hypersensitive to mifepristone compared with PR-B.

**Conclusions:**

Taken together with previous mechanistic studies, the findings provide clinical evidence in support of the role of PR-A in L-BCa metastasis. They also suggest the possibility of developing selective PR-A modulators for future interventions in appropriate clinical situations.

## Background

Breast oncogenesis may occur over decades. The majority (> 78%) of initial breast cancer diagnoses are in women over 50 years of age [1] and median age, 61 years [2]. The majority of breast cancer (BCa) cases are of the luminal subtype (L-BCa), which is estrogen receptor (ER)-positive [3] and continues to express ER into advanced stages [4]. Primary L-BCa is exquisitely sensitive to anti-estrogens. Unfortunately, L-BCa has frequently metastasized prior to diagnosis and the metastatic tumors may also appear long after cessation of therapy [5, 6]. At least 20% of BCa patients have ER+ tumors showing distal metastasis [7]. Metastatic L-BCa is typically incurable. Our previous studies have mechanistically linked invasiveness and metastasis of L-BCa to a novel hormonal mechanism [8, 9].

Growth of ER+ breast tumors is supported by estrogen but the hormone suppresses tumor invasiveness, regardless of hormone sensitivity of growth and suppresses tumor progression [10–16]. In contrast, progestins support invasiveness and metastasis in L-BCa cells [17, 18]. A single gene encodes two isoforms of the progesterone receptor (PR), PR-A and PR-B, via alternative promoter usage. PR-B only differs from PR-A in having an amino-terminal segment of 164 residues containing the activation function, AF3 [19]. Despite overlap in the gene regulatory patterns of PR-B and PR-A, the two isoforms also show a clear distinction in terms of the strength of this regulation and also in the target genes that they activate or repress [19–21]. The two PR isoforms can heterodimerize and regulate a small set of unique genes [20, 22]. PR-A and PR-B show similar levels of expression in normal breast but breast oncogenesis is associated with loss of this coordinate expression, mostly resulting in a high PR-A:PR-B ratio, in early as well as progressed lesions [23]. The higher ratio of PR-A to PR-B is generally due to PR-A overexpression and has been reported to be negatively associated with disease free survival (DFS), although the mechanistic underpinnings were not established [24].

The pre-menopause plasma estrogen level is 1.4 nM − 1.6 nM (follicular phase), and 3.6 nM - 4.2 nM (luteal phase) [25]. Progesterone levels in the plasma range from < 4 nM in the follicular phase, up to > 50 nM in the luteal phase [26]. After menopause, the circulating hormone levels show a marked decline (median value for estrogen of 0.14 nM and median value for progesterone of 0.13 nM), but hormone levels of up to ~ 1 nM may be retained in breast tissue [27, 28]. Previous in vitro studies in the literature had suggested progesterone induces invasiveness of BCa cells principally via PR-B [29, 30] although clinical observations noted more frequent elevation of PR-A in ductal carcinoma in situ (DCIS) and invasive breast lesions [23] and associated PR-A rather than PR-B with lower DFS [24]. Notably, those in vitro studies were conducted using relatively high progesterone concentrations (corresponding to luteal stage, pregnancy or pharmacological plasma levels) and excluded estrogen signaling [29–33].

We have reported studies covering the full ranges of pre-menopausal and post-menopausal levels of estrogen and progesterone, conducted with both estrogen and progesterone signaling present, using T47D, ZR-75-1 and BT474 cells as well as isogenic recombinant T47D cells expressing a single PR isoform [8]. Previous studies of the effects of high dose progesterone on invasion and metastasis utilized models in which estrogen signaling was absent. Therefore we took into account the possibility that cross-talk with estrogen signaling may influence regulation of invasiveness by progestins at physiological hormone levels. At concentrations < 0.01 nM, estrogen strongly suppressed invasiveness of ER+ BCa cells. At relatively low concentrations (< 1 nM), progestins abrogated inhibition of invasiveness caused by estrogen. It took relatively higher concentrations of progestins, (in the range of 5 nM - 50 nM) to induce invasiveness in the absence of estrogen, in a progestin dose-dependent manner. It was the PR-A isoform that mediated rescue of invasiveness from estrogen regulation by progesterone and this effect was uninfluenced by PR-B [8]. In contrast, estrogen-independent induction of invasiveness by progestin was mediated by PR-B and this occurred at either pregnancy-associated or pharmacological levels of progestin [8]. When PR-A was 2–3 fold overexpressed in PR-A+/PR-B+ cells, a lower concentration of progestin (< 0.2 nM) completely rescued invasiveness. These hormonal effects on invasiveness were independent of HER2 status. Thus progesterone supports BCa cell invasiveness by counteracting estrogen, acting exclusively through PR-A, at hormone concentrations corresponding to the full physiological ranges of estrogen and progesterone levels, both prior to and after menopause [8].

To interrogate the role of the ER-PR-A crosstalk in metastasis, we investigated selective crosstalk mechanisms of PR-A with ER via microRNAs (miRNAs), which have an extensive role in estrogen signaling [9]. Among miRNAs regulated by estrogen or progesterone, miR-92a-3p and miR-26b-5p mediated the PR-A–ER crosstalk. Progesterone plus PR-A suppressed induction of miR-92a-3p and repression miR-26b-5p by estrogen. This resulted in regulation of genes involved in invasiveness and metastasis and also completely rescued invasiveness in vitro. In murine xenograft models, when miR-92a-3p was constitutively expressed or when miR-26b-5p was inhibited, metastasis was profoundly suppressed, similar to knockdown of PR [9]. Notably, these two miRNAs did not regulate each other and, based on quantitative changes in mRNAs of genes involved in invasion and/or metastasis using a commercially available pathway cDNA array, we found that the two micro RNAs mediate independent hormone-regulated pathways controlling invasion and metastasis that are convergent. Therefore, the strong suppression of invasiveness by even very low concentrations of estrogen (~ 0.01 nM) may be the net effect of sub-optimal regulation of these two miRNAs by estrogen. In primary tumors, PR-A expression showed negative correlation with miR-92a-3p and positive correlation with miR-26b-5p, but the strength of this correlation was moderate, as expected because of variable stromal content in resected tumor specimens, the likely influence of variability in the range of plasma progesterone levels in postmenopausal women and differences in expression of a compliment of coregulators and downstream effectors. The mechanistic studies suggest that hormonal crosstalk of PR-A with ER enables metastasis of luminal BCa, notably by opposing regulation of critical microRNAs by estrogen. This occurs even at hormone levels corresponding to plasma hormone status in the follicular stage and post-menopause.

Public RNAseq databases on the transcriptome profiles of breast tumors, such as The Cancer Genome Atlas (TCGA), do not distinguish PR-A, because it has multiple transcripts arising from alternative promoter usage that only differ from the PR-B transcript with respect to the positions of their 5′ ends. Therefore, in this study, we undertook a prospective analysis to clinically validate the role of PR-A in metastasis. We further investigated the possibility of isoform-selective modulation of PR-A.

**Fig. 1:**
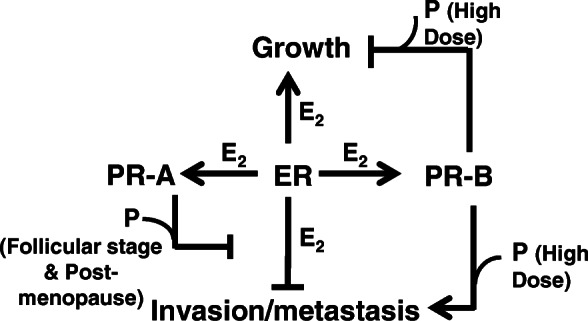
Schematic showing cross-talk between estrogen (E2) and progesterone (P) signaling in regulating progression of breast cancer

As described above, previous studies have rigorously elucidated a mechanistic model of breast cancer metastasis that should predict the manner in which expression of ER, PR-A and PR-B are associated with L-BCa metastasis (Fig. 1). In this model, PR-A is induced by estrogen and thus ER limits its own ability to suppress invasion and metastasis. Additionally, PR-A may also be further up-regulated in L-BCa via mechanisms that are independent of estrogen. PR-B on the other hand requires relatively high physiological levels of progesterone (premenopausal luteal stage and pregnancy associated levels) to induce metastasis. Therefore, the model predicts a strong clinical association of PR-A with metastasis of primary L-BCa but, at best, a weak association of PR-B with metastasis. The model also predicts that the association of PR-A with metastasis should be uninfluenced by the relative expression of PR-B as PR-B influences metastasis by a separate mechanism and only at high progesterone levels. At the same time the model predicts a likely negative association of ER with metastasis. The mechanistic studies also predict that expression of HER2 will not affect the association of PR-A with metastasis.

## Methods

### Cell line models, tissue specimens and reagents

Isogenic T47D-A (ER+/PR-A+/PR-B-null) and T47D-B (ER+/PR-B+/PR-A-null) recombinant cells were a generous gift from Dr. Katherine Horowitz (University of Colorado, Denver, CO) and were cultured as described previously All human tumor samples, from female patients, classified as treatment naïve ER+/PR+ primary breast carcinoma were obtained from the Cooperative Human Tissue Network (CHTN). For each tumor tissue selected, the largest dimension was in the range of 1.5 cm–2 cm. Lipofectamine 2000 was from Thermo Scientific (product number 78410). R5020, and RU486 (mifepristone) were purchased from Sigma Aldrich (Saint Louis,MO).

### Isolation and measurement of mRNA

Total RNA was isolated from tissues using the Purelink RNA Mini Kit (ThermoFisher Scientific, Grand Island, NY). Breast tumor tissue lysates were prepared by suspending 30 mg of tissue in 500 μl of the lysis buffer and homogenized using the PRO200 tissue homogenizer (catalog no. 01–01200, BioGen) for 15 s on ice. Homogenized solution was centrifuged at high, 15,000 x g, and supernatant was used for RNA extraction. Reverse-transcription PCRs were performed using high-capacity complementary DNA archive kit (Life Technologies, Inc., Carlsbad, CA). cDNA was measured by quantitative real-time RT-PCR using TaqMan probes and the StepOne Plus real-time PCR system (Life Technologies). All RNA measurements were performed in biological triplicates, and all Ct values were normalized to intrasample GAPDH (mRNA).

### Statistical methods

A total of 125 breast cancer tumor tissues (extracted from 125 patients) were analyzed by real-time quantitative PCR (qPCR) to measure Ct values of Total PR, PR-A and PR-B. The qPCR was repeated 3 times for each RNA to generate biological replicates, resulting in 375 Ct values for each RNA. Note that the Ct value is defined as the number of cycles needed for the fluorescence to reach a specific threshold level of detection and is reciprocally correlated with the amount of template present in the reaction [34]. The efficiency of each primer pair (E = 2) was used to normalize the qPCR data and a relative gene expression value with regard to control was calculated using the equation ${2}^{\varDelta {CT}_{sample}}={2}^{Ct_{control}-{Ct}_{sample}}$, where *Ct*_*control*_ is the Ct value for a housekeeping gene and *Ct*_*sample*_ is the Ct value for each amplification. The relative gene expression value of PR-A was obtained by the equation ${2}^{{\varDelta C t}_{Total\  PR}}-{2}^{\varDelta C{t}_{PR-B}}$. In particular, if *ΔCt*_*Total PR*_ < *ΔCt*_*PR* − *B*_, we assigned zero for the relative expression vale of PR-A [35]. To calculate the correlation coefficients in repeated measures [36] among RNAs, a linear mixed-effect modeling was used after each relative gene expression value was transformed with the Box-Cox transformation with two parameters due to zeros [37]. In addition, the associations between the lymph node status (positive vs. negative) and each of the expression levels of Total PR, PR-A and PR-B were examined using those 125 tumor tissues. In order to take into account three replicates in each measurement, a linear mixed-effects model was used to assess the associations. All statistical analyses were performed using the statistical software package R (https://www.r-project.org) and the Box-Cox transformation with two parameters were carried out using the function boxcoxfit available in the R package geoR.

### Other experimental methods

Transient transfections and luciferase reporter assays have been described [38]. IC_50_ values for mifepristone were determined using GraphPad Prism dose-response analysis of the log-transformed drug concentrations and response.

## Results

### Prospective investigation of hormone receptor expression in relation to metastasis of L-BCa

The most reliable indicator of metastasis of a primary luminal breast tumor is nodal involvement, assessed primarily from axillary lymph node biopsies, especially sentinel node biopsies [39]. The inherent metastatic potential of a tumor, which is independent of treatment, determines the frequency and extent of tumor spread in the years spanning the time of occurrence of the oncogenic event and the initial cancer diagnosis as well as the years following cessation of treatment. Therefore, we used axillary lymph node status at time of diagnosis as an objective indicanaivtor of metastatic potential of the primary tumors to investigate whether PR-A expression in the tumors is associated with their ability to metastasize, independent of treatment.

We have previously reported that in ER+ primary BCa tumor specimens the relative protein levels of PR-A and PR-B are reflected by their relative mRNA levels [9]. However, public datasets on the transcriptome profiles of breast tumors, such as TCGA, do not distinguish PR-A from PR-B, because it has multiple transcripts arising from alternative promoter usage that only differ from the PR-B transcript with respect to the positions of their 5′ ends. Therefore, we prospectively examined treatment-naïve primary ER+ breast tumors from 125 patients, using a standardized differential quantitative RT-PCR assay for PR-A and PR-B. The patient baseline characteristics are described in Table 1. They include similar numbers of patients with vs. without node involvement at time of diagnosis with mostly ductal carcinomas and lesions ranging from Grade I – Grade III. The tumors were predominantly HER2-negative. In these specimens, we examined expression of mRNAs for PR-A, PR-B and ER.

**Table 1:** Patient baseline characteristics. The comparisons were conducted by Fisher’s exact tests for categorical variables and by Kruskal-Wallis tests for continuous variables

	Lymph node status		All (***N*** = 125)	p
	Negative (***N*** = 62)	Positive (***N*** = 63)		
**Age - median (range)**	59.5 (25–87)	54 (35–87)	57 (25–87)	0.054
**Histologic type - no. (%)**				0.612
Ductal	45 (73)	40 (63)	85 (68)	
Lobular	9 (15)	15 (24)	24 (19)	
Mixed	5 (8)	5 (8)	10 (8)	
None	3 (5)	3 (5)	6 (5)	
**Grade - no. (%)**				0.566
I	10 (16)	7 (11)	17 (14)	
II	27 (44)	26 (41)	53 (42)	
III	24 (39)	30 (48)	54 (43)	
missing	1 (2)	0 (0)	1 (1)	
**HER2 status - no. (%)**				0.572
Negative	46 (74)	50 (79)	96 (77)	
Equivocal	9 (15)	5 (8)	14 (11)	
Positive	3 (5)	2 (3)	5 (4)	
missing	4 (6)	6 (10)	10 (8)	

### Correlation of PR-A expression with those of total PR, PR-B, ER and with node status

**Fig. 2:**
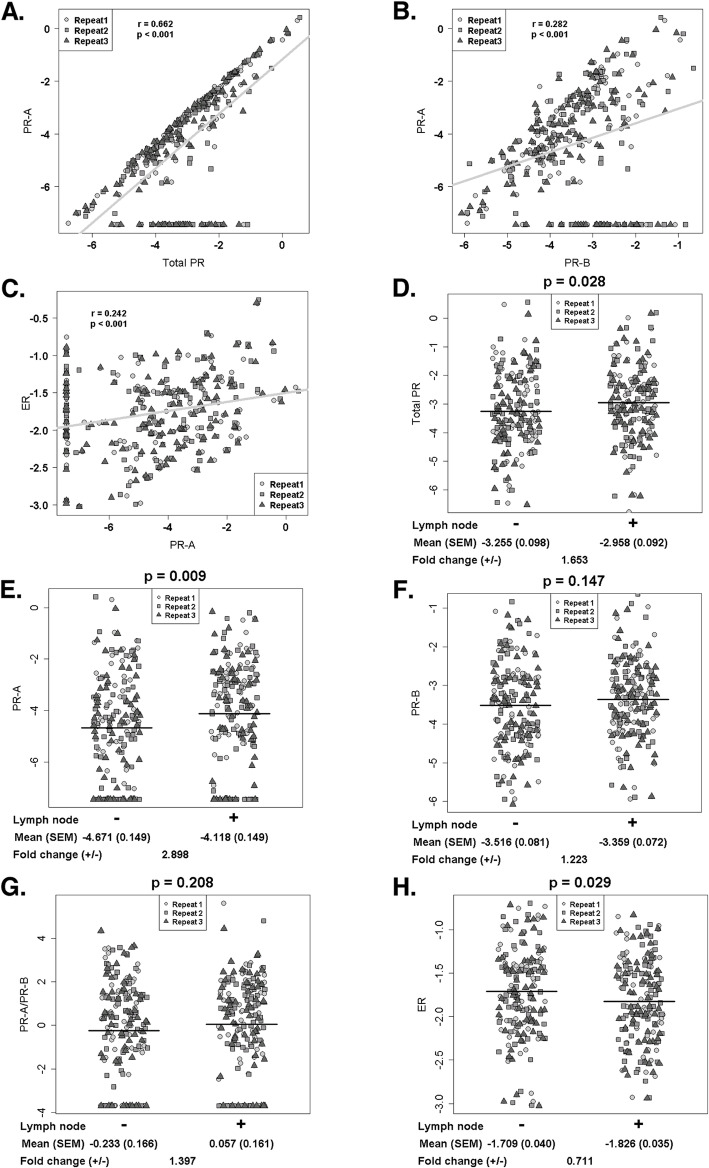
Relationships among total PR, PR-A, PR-B and ER and their associations with lymph node status. Scatter plots of total PR vs. PR-A (Panel **a**), PR-B vs. PR-A (Panel **b**), and ER vs. PR-A (Panel **c**). In Panels A-C, the relative gene expression values are represented after Box-Cox transformation. The solid line represents the linear regression line. The correlation coefficients (r) in repeated measures were estimated using linear mixed-effects models. Associations between lymph node status versus total PR (Panel **d**), PR-A (Panel **e**), PR-B (Panel **f**), PR-A/PR-B (Panel **g**), and ER (Panel **h**). In Panels D-H, the relative gene expression values are represented after Box-Cox transformation. The solid lines represent the overall mean values. The associations in repeated measures were assessed using a linear mixed-effects model. The transformed expression levels in each group were summarized by mean and standard error of mean (SEM). The fold change (lymph node positive vs. negative) was calculated using the medians of raw expression levels

PR-A expression correlated moderately with total PR (r value, 0.66) and weakly with expression of PR-B (r value, 0.28) or ER (r value, 0.24) (Fig. 2a**-c**). In the unadjusted analysis, total PR (p, 0.028) and PR-A (p, 0.009) showed a significant (positive) association with node involvement with 1.7- and 2.9-fold higher median values vs. node negative tumors, respectively (Fig. 2d, e and Table 2). PR-B was not significantly correlated with node status and neither was the ratio of PR-A to PR-B (Fig. 2f, g and Table 2). In contrast, in the unadjusted analysis, ER notably showed a negative correlation with node involvement (p, 0.029) (Fig. 2h and Table 2).

**Table 2:** Associations with lymph node status. The univariable analysis shows the associations between each RNA and lymph node status, while the multivariable analysis presents the associations after adjusting the effects of age, histology, and grade. All the analyses were carried out using linear mixed-effect models. The estimate and SE represent the estimated slope and its associated standard error of lymph node positive

	Univariable analysis			Multivariable analysis		
	Estimate (SE)	t-value	p	Estimate (SE)	t-value	p
**Total PR**	0.297 (0.135)	2.205	***0.028***	0.315 (0.137)	2.305	***0.022***
**PR-B**	0.157 (0.108)	1.453	0.147	0.154 (0.110)	1.401	0.162
**PR-A**	0.553 (0.210)	2.626	***0.009***	0.639 (0.214)	2.994	***0.003***
**ER**	−0.116 (0.053)	−2.194	***0.029***	−0.082 (0.051)	−1.593	0.112
**PR-A/PR-B**	0.291 (0.231)	1.260	0.208	0.408 (0.234)	1.742	0.082

When the data was adjusted by multivariable analysis for age, histology and grade, PR-A showed the strongest association with node positivity (t-value, 2.99; p, 0.003) (Table 2). Total PR showed a similar trend but neither PR-B nor the PR-A/PR-B ratio correlated significantly with node status. Further, adjusting to remove the effect of PR-B did not significantly affect the relationship between PR-A and node status in the univariable or multivariable analysis (Supplementary Table 1). The adjusted analysis weakened the negative correlation of ER with node involvement.

The ability of PR-A to predict metastasis held true when its expression level was dichotomized (low vs. high) by its median value, with OR (95% CI) of 2.3 (1.5–3.5) (*p* < 0.001) in the multivariable logistic regression analysis that adjusted for age, histology and grade (Supplementary Table 2). Whereas total PR showed a similar trend, PR-B, the PR-A/PR-B ratio and ER did not predict metastasis (Supplementary Table 2). Moreover, adjusting to remove the effect of PR-B did not influence the predictive ability of PR-A, either in the univariable or multivariable analysis (Supplementary Table 3).

Our study cohort had a limited number of HER2-positive tumor specimens, reflecting the relatively low frequency of HER2 amplification in L-BCa. Specifically, the study included 96 HER2-negative specimens, 5 HER2-positive specimens, 14 specimens with equivocal HER2 status and 10 specimens in which data on HER2 expression was unavailable. Therefore, we repeated the univariable and multivariable analysis using specimens that were classified either as HER2-negative (96 specimens) or HER2-non-negative (5 + 14 = 19 specimens). Including HER2 in this manner in the adjusted analysis did not alter the association of PR-A with node involvement (Supplementary Table 4). The house keeping gene used in all cases was GAPDH. The intra-sample Ct values for GAPDH were used to normalize for variabilities in the exact amount of RNA in each assay as well as possible variability in the efficiency of reverse transcription between samples or across experiments. We did not see an association between the raw GAPDH Ct values and node status or other tumor characteristics (Supplementary Figure 1).

### Selective antagonism of isoform A of PR

**Fig. 3:**
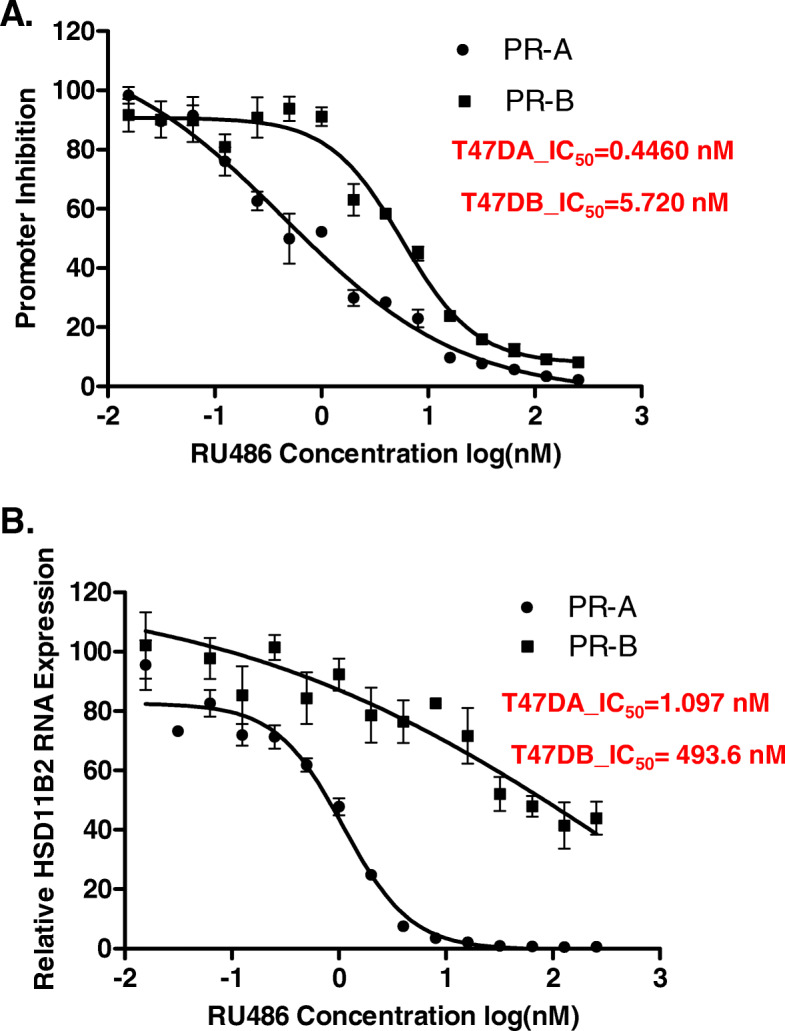
Mifepristone dose sensitivity for inhibition of the transcriptional activity of PR-A compared with PR-B. In Panels **a**, hormone depleted T47D-A and T47D-B cells were transfected with a plasmid expressing a minimal promoter-luciferase reporter containing a progesterone responsive element (PRE-TATA-LUC) and simultaneously treated with vehicle (DMSO) or 10 nM R5020 (P) plus varying concentrations of RU486 (0.015625 nM- 256 nM) for 48 h. Cells were harvested by preparing lysates for measurement of luciferase activity. In Panel **b**, T47D-A and T47D-B cells were similarly treated but without transfection. At the end of the treatment, total RNA was isolated and mRNA for the HSD11B2 gene was measured by quantitative RT-PCR. For Panels **a** and **b**, error bars have been included to denote SEM for 3 replicates

As PR-A is likely functionally associated with L-BCa metastasis over a much broader range of physiological hormone status than PR-B, it was of interest to seek proof-of-principle for whether it would be possible to selectively modulate the PR-A isoform as a potential future strategy for intervention in L-BCa metastasis. As studies have shown that administering low dose mifepristone for extended periods is well tolerated in humans and as our previous studies noted that a low (1 nM) concentration of mifepristone only inhibited the effect of PR-A but not PR-B on invasiveness of L-BCa cells, we examined the relative sensitivities of the transcriptional activities of PR-A and PR-B to mifepristone. In a hormone-dependent promoter activation assay using isogenic T47-D cells expressing either PR-A alone (T47D-A cells) or PR-B alone (T47D-B cells), PR-A was hyper-sensitive to mifepristone compared with PR-B, with IC50 values for the drug of 0.45 × 10^− 9^ M and 5.72 × 10^− 9^ M respectively, for PR-A and PR-B (Fig. 3a). We then used the same isogenic cells to examine the mifepristone dose-dependence for the two PR isoforms for inhibition of activation of HSD11B2, a known common endogenous gene target of PR-A and PR-B [39] Again, hormone-dependent induction of the HSD11B2 mRNA by PR-A was highly sensitive to mifepristone, which was required at a much higher dose to inhibit PR-B (Fig. 3b).

## Discussion

Diagnostic evaluation of PR status in primary breast cancer does not typically distinguish PR isoforms. However, two major clinical studies have suggested an association between PR-A and progression of L-BCa. In one study, coordinate expression of PR-A and PR-B observed in normal breast epithelial cells and in benign breast tumors was reportedly frequently disrupted in both DCIS and in invasive lesions, where a PR-A: PR-B ratio > 1 was more frequent than a ratio < 1 [23]. As the extent of dysregulated expression varied from cell to cell within the lesions, the authors suggested that loss of coordinate expression of the PR isoforms is an early event in the development of malignancy with potential implications in tumor progression [23]. Another study conducted using axillary node-positive breast tumors noted that an elevated PR-A: PR-B ratio was more frequently the result of overexpression of PR-A and that patients harboring primary tumors with higher PR-A: PR-B ratios were ~ 2.8 – fold more likely to relapse [24]. Our more recent in vitro mechanistic and pre-clinical model studies have rigorously established that PR-A causes invasiveness and metastasis in response to progesterone levels in its pre- and post-menopausal ranges by counteracting the effect of estrogen [8, 9]. Moreover, elevated expression of PR-A hyper-sensitizes the receptor to progesterone levels at the low end of its post-menopausal range [8, 9]. We have previously established and reported that in primary ER+ breast cancer tumors, the mRNA levels of PR-A and PR-B correlate with their respective protein levels [9]. The present study directly links this mechanistic role of PR-A with metastasis of L-BCa in a patient population while offering a basis for the previous clinical observations noted above.

The distinctive relationships of the expression levels of PR-A, PR-B and ER in primary L-BCa lesions with node involvement is entirely consistent with predictions based on the previously established mechanistic model (Fig. 1) in which PR-A and PR-B have independent roles in promoting invasiveness and metastasis, with PR-A alone being responsive to the entire range of pre- and post-menopausal progesterone levels, functioning by counteracting the influence of estrogen/ER. As predicted from the model, PR-A was strongly associated with node involvement both in a univariable analysis and after adjusting for age, pathologic type, tumor grade and PR-B. In contrast, PR-B did not show an association with node involvement. ER actually showed a negative correlation with node involvement in the univariable analysis but in the multivariable analysis, this association was weaker, suggesting a more complex role for ER in the pathophysiology of L-BCa. The patients included in this study had predominantly HER2-negative tumors and there was no apparent influence of HER2 on the association of PR-A with node involvement, consistent with the previous mechanistic studies.

Together with previous studies, this study supports the view that progesterone, acting largely through PR-A, contributes to metastasis of L-BCa. However, even in post-menopausal women, it would not be feasible to completely inhibit the actions of progesterone to suppress invasion and metastasis of L-BCa. This is because in these women progesterone is needed to check proliferative effects of estrogen on the uterus. In mice in which the PR-A isoform was selectively knocked out, estrogen and even progesterone (acting via PR-B) caused hyperplasia of the uterine epithelium [40]. However, this rodent model data on the physiological role of PR-A in the uterus do not necessarily appear to be able to predict the likely side effects of pharmacologically selectively modulating PR-A in humans to suppress metastasis of L-BCa, when the following observations are considered together. First, we noted in this study that the general transcriptional activities of PR-A and PR-B are differentially sensitive to mifepristone, with PR-A being inhibited in a much lower dose range of mifepristone than PR-B. Second, as previously reported by us, a low dose (1 nM) of mifepristone completely inhibited the ability of PR-A to rescue invasiveness of L-BCa cells from suppression by estrogen [8]. In contrast, under the same conditions, mifepristone had no effect on the estrogen-independent induction of invasiveness by PR-B [8]. Finally, in (premenopausal) women treated long-term daily with low dose mifepristone as a potential contraceptive, endometrial hyperplasia was not a significant concern (41). It is therefore possible that in humans, PR-A is selectively targeted by low dose mifepristone, with minimal side effects on the uterus. From a mechanistic standpoint, hormone-dependent transcriptional activities of PR-A and PR-B are quite distinct from each other both in their patterns of regulation of mRNAs [19–21] and micro RNAs [9]. There is a clear structural basis for this functional difference between the PR isoforms, i.e., the amino-terminal 164 residues of PR-B that is lacking in PR-A. Therefore it should be possible in principle to develop selective modulators of PR-A that could potentially offer an effective means of intervention in progression of L-BCa in certain patient groups such as post-menopausal women at high risk.

## Conclusion

Taken together, the current clinical study, our previous detailed mechanistic studies and other related studies in the literature strongly support the view that, throughout a woman’s pre- and post-menopausal years, PR-A is a major driver in promoting invasiveness and metastasis of L-BCa by suppressing specific aspects of estrogen/ER action. The studies also suggest that PR-A isoform specific modulation is achievable as a possible intervention for progression of L-BCa in clinically appropriate situations.

## Supplementary information

**Additional file 1: Table S1.** The univariable analysis shows the association between PR-A and lymph node status and the multivariable analysis shows the associations after adjusting the effects of PR-B along with age, histology, and grade. All the analyses were carried out using linear mixed-effect models. The estimate and SE represent the estimated slope and its associated standard error of lymph node positive. **Table S2.** The expression levels were dichotomized by the median values for each RNA (Low vs. High). The univariable analysis shows the associations between each RNA and lymph node status, while the multivariable analysis presents the associations after adjusting the effects of age, histology, and grade. All the analyses were carried out using logistic mixed-effect models. OR and CI stand for ‘odds ratio’ and ‘confidence interval’. **Table S3.** The expression levels were dichotomized by the median values for each RNA (Low vs. High). The univariable analysis shows the associations between PR-A and lymph node status, while the multivariable analysis presents the associations after adjusting the effects of PR-B along with age, histology, and grade. All the analyses were carried out using logistic mixed-effect models. OR and CI stand for ‘odds ratio’ and ‘confidence interval’. **Table S4.** Associations with lymph node status. The univariable analysis shows the associations between each RNA and lymph node status, while the multivariable analysis presents the associations after adjusting the effects of age, histology, grade, and HER2 status. All the analyses were carried out using linear mixed-effect models. The estimate and SE represent the estimated slope and its associated standard error of lymph node positive.

**Additional file 2: Figure S1.** The distribution of GAPDH Ct values **(a)** and the associations between GAPDH Ct value and each of lymph node **(b**), histologic type **(c)**, and grade **(d)**. In **(c)-(d)**, the horizontal bars indicate a mean value and the *p*-value was calculated using repeated measures two-sample t-test and one-way ANOVA under linear mixed-effects models.

## Data Availability

Data and plasmid constructs from this study will be made available upon request, following institutional guidelines of Wayne State University.

## Abbreviations

L-BCa: Luminal breast cancer
PR: Progesterone receptor
PR-A: Progesterone receptor A
PR-B: Progesterone receptor B
ER: Estrogen receptor
DFS: Disease free survival
DCIS: Ductal carcinoma in situ
miRNA: Micro RNA
TCGA: The Cancer Genome Atlas
CHTN: Cooperative Human Tissue Network
qPCR: Real-time quantitative PCR
