# Supplementary material for: Clinical association of progesterone receptor isoform A with breast cancer metastasis consistent with its unique mechanistic role in preclinical models
Source: BMC Cancer. 2020 Jun 3;20:512. doi: 10.1186/s12885-020-07002-0 (PMC7268268; doi:10.1186/s12885-020-07002-0)
Supplement: Supplementary file 1 — Additional file 1: Table S1. The univariable analysis shows the association between PR-A and lymph node status and the multivariable analysis shows the associations after adjusting the effects of PR-B along with age, histology, and grade. All the analyses were carried out using linear mixed-effect models. The estimate and SE represent the estimated slope and its associated standard error of lymph node positive. Table S2. The expression levels were dichotomized by the median values for each RNA (Low vs. High). The univariable analysis shows the associations between each RNA and lymph node status, while the multivariable analysis presents the associations after adjusting the effects of age, histology, and grade. All the analyses were carried out using logistic mixed-effect models. OR and CI stand for ‘odds ratio’ and ‘confidence interval’. Table S3. The expression levels were dichotomized by the median values for each RNA (Low vs. High). The univariable analysis shows the associations between PR-A and lymph node status, while the multivariable analysis presents the associations after adjusting the effects of PR-B along with age, histology, and grade. All the analyses were carried out using logistic mixed-effect models. OR and CI stand for ‘odds ratio’ and ‘confidence interval’. Table S4. Associations with lymph node status. The univariable analysis shows the associations between each RNA and lymph node status, while the multivariable analysis presents the associations after adjusting the effects of age, histology, grade, and HER2 status. All the analyses were carried out using linear mixed-effect models. The estimate and SE represent the estimated slope and its associated standard error of lymph node positive. [file 12885_2020_7002_MOESM1_ESM.pptx]

## Slide 1
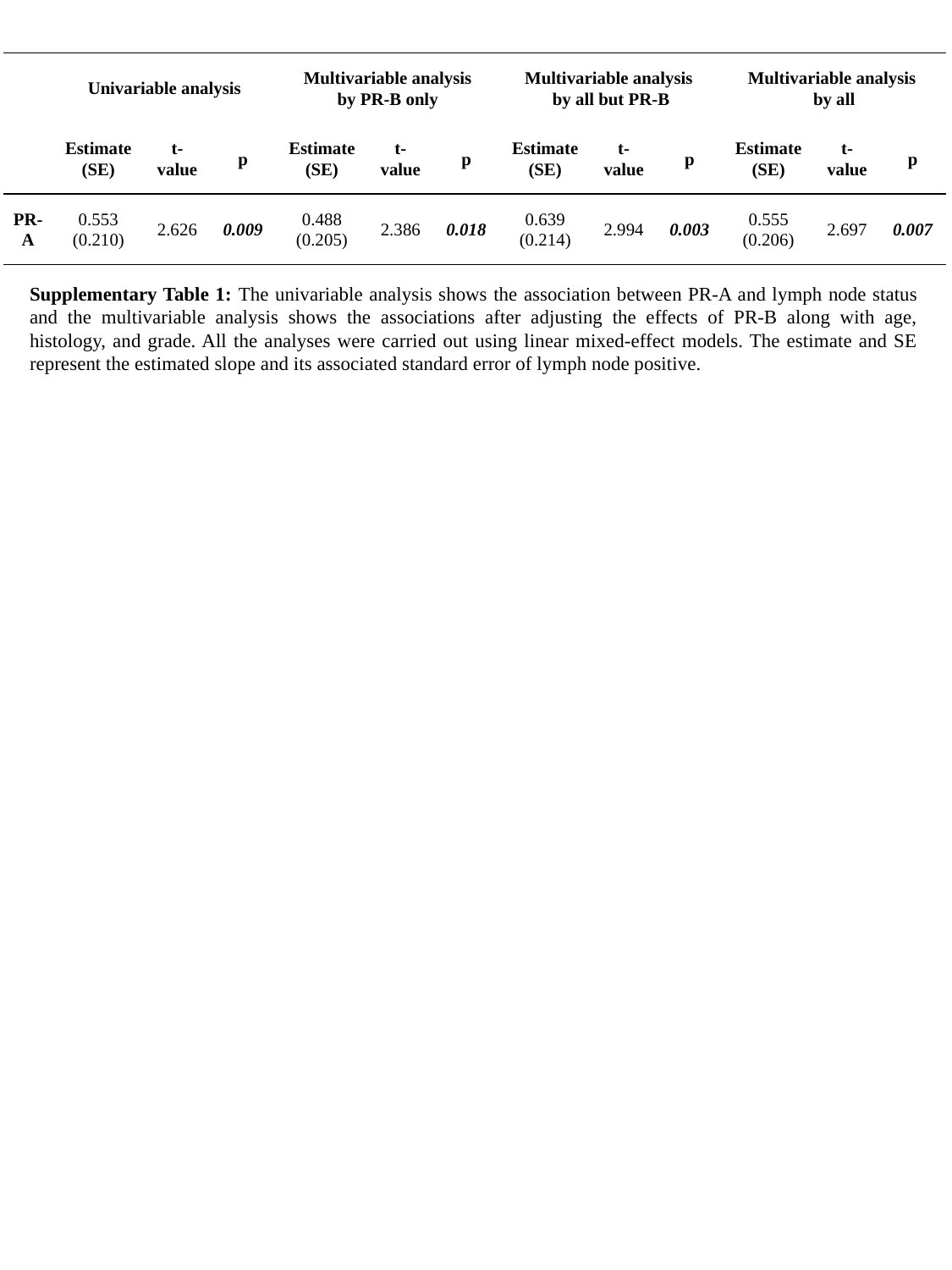

| | Univariable analysis | | | Multivariable analysis by PR-B only | | | Multivariable analysis by all but PR-B | | | Multivariable analysis by all | | |
| --- | --- | --- | --- | --- | --- | --- | --- | --- | --- | --- | --- | --- |
| | Estimate (SE) | t-value | p | Estimate (SE) | t-value | p | Estimate (SE) | t-value | p | Estimate (SE) | t-value | p |
| PR-A | 0.553 (0.210) | 2.626 | 0.009 | 0.488 (0.205) | 2.386 | 0.018 | 0.639 (0.214) | 2.994 | 0.003 | 0.555 (0.206) | 2.697 | 0.007 |
Supplementary Table 1: The univariable analysis shows the association between PR-A and lymph node status and the multivariable analysis shows the associations after adjusting the effects of PR-B along with age, histology, and grade. All the analyses were carried out using linear mixed-effect models. The estimate and SE represent the estimated slope and its associated standard error of lymph node positive.

## Slide 2
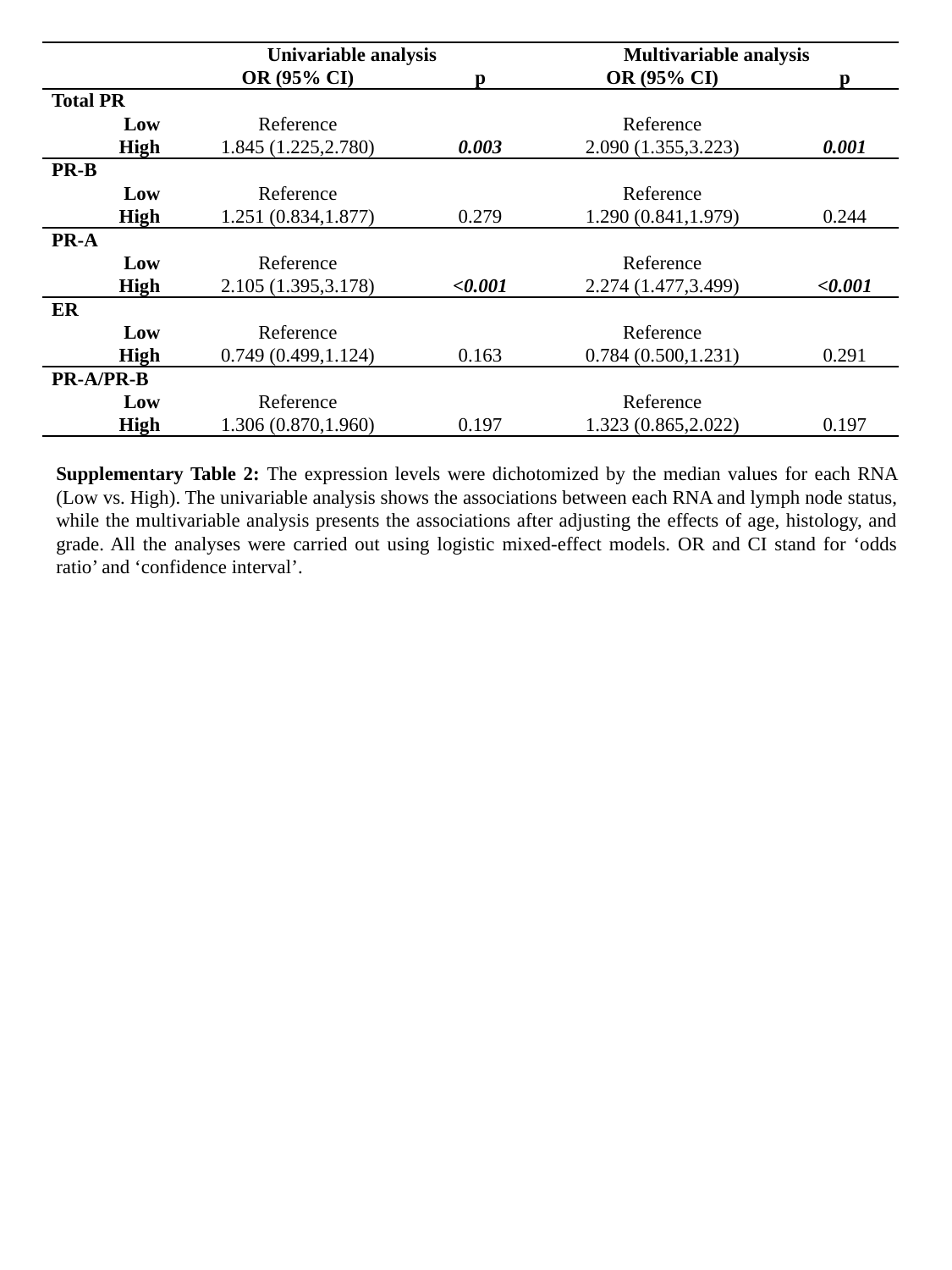

| | Univariable analysis | | Multivariable analysis | |
| --- | --- | --- | --- | --- |
| | OR (95% CI) | p | OR (95% CI) | p |
| Total PR | | | | |
| Low | Reference | | Reference | |
| High | 1.845 (1.225,2.780) | 0.003 | 2.090 (1.355,3.223) | 0.001 |
| PR-B | | | | |
| Low | Reference | | Reference | |
| High | 1.251 (0.834,1.877) | 0.279 | 1.290 (0.841,1.979) | 0.244 |
| PR-A | | | | |
| Low | Reference | | Reference | |
| High | 2.105 (1.395,3.178) | <0.001 | 2.274 (1.477,3.499) | <0.001 |
| ER | | | | |
| Low | Reference | | Reference | |
| High | 0.749 (0.499,1.124) | 0.163 | 0.784 (0.500,1.231) | 0.291 |
| PR-A/PR-B | | | | |
| Low | Reference | | Reference | |
| High | 1.306 (0.870,1.960) | 0.197 | 1.323 (0.865,2.022) | 0.197 |
Supplementary Table 2: The expression levels were dichotomized by the median values for each RNA (Low vs. High). The univariable analysis shows the associations between each RNA and lymph node status, while the multivariable analysis presents the associations after adjusting the effects of age, histology, and grade. All the analyses were carried out using logistic mixed-effect models. OR and CI stand for ‘odds ratio’ and ‘confidence interval’.

## Slide 3
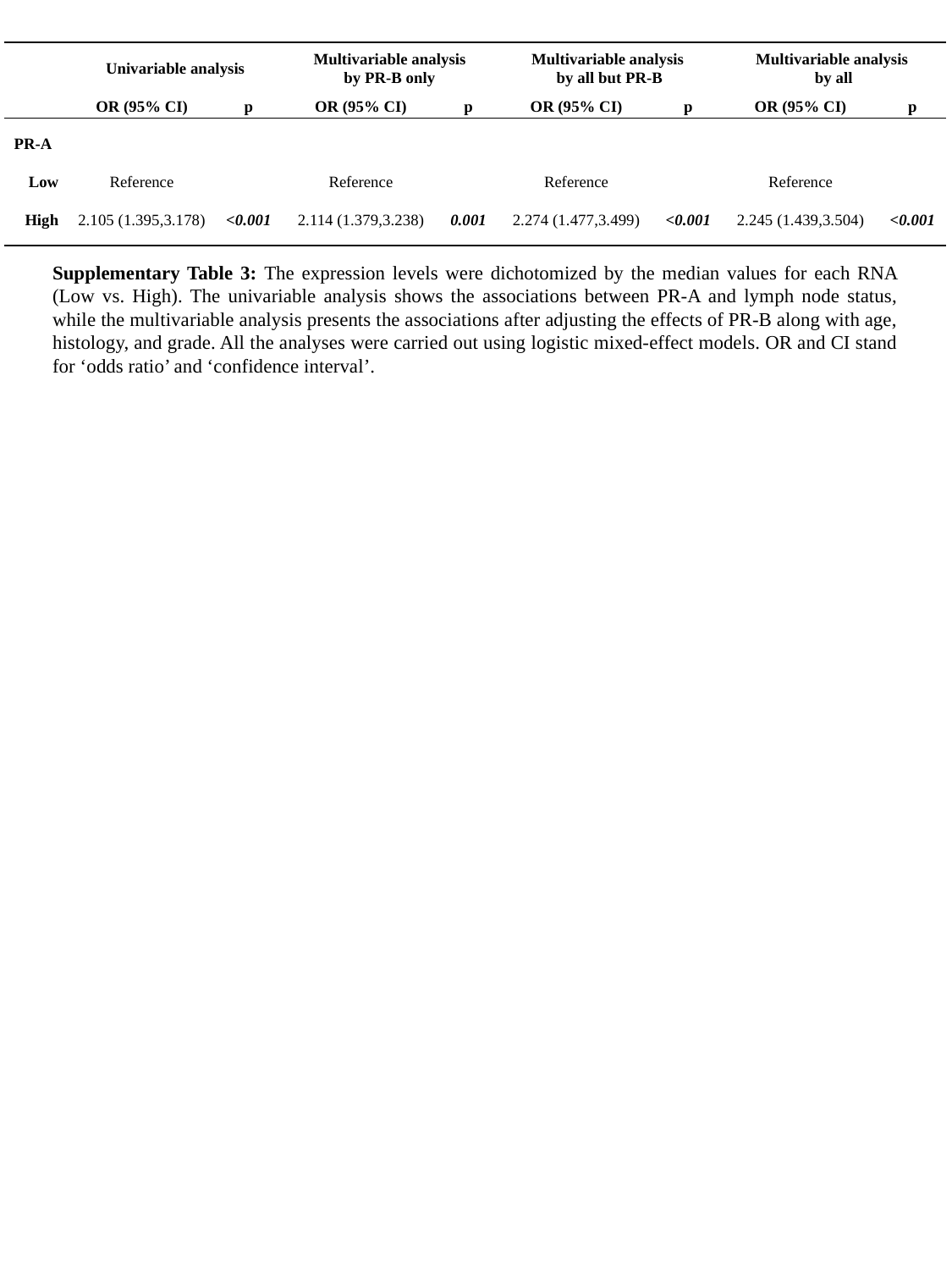

| | Univariable analysis | | Multivariable analysis by PR-B only | | Multivariable analysis by all but PR-B | | Multivariable analysis by all | |
| --- | --- | --- | --- | --- | --- | --- | --- | --- |
| | OR (95% CI) | p | OR (95% CI) | p | OR (95% CI) | p | OR (95% CI) | p |
| PR-A | | | | | | | | |
| Low | Reference | | Reference | | Reference | | Reference | |
| High | 2.105 (1.395,3.178) | <0.001 | 2.114 (1.379,3.238) | 0.001 | 2.274 (1.477,3.499) | <0.001 | 2.245 (1.439,3.504) | <0.001 |
Supplementary Table 3: The expression levels were dichotomized by the median values for each RNA (Low vs. High). The univariable analysis shows the associations between PR-A and lymph node status, while the multivariable analysis presents the associations after adjusting the effects of PR-B along with age, histology, and grade. All the analyses were carried out using logistic mixed-effect models. OR and CI stand for ‘odds ratio’ and ‘confidence interval’.

## Slide 4
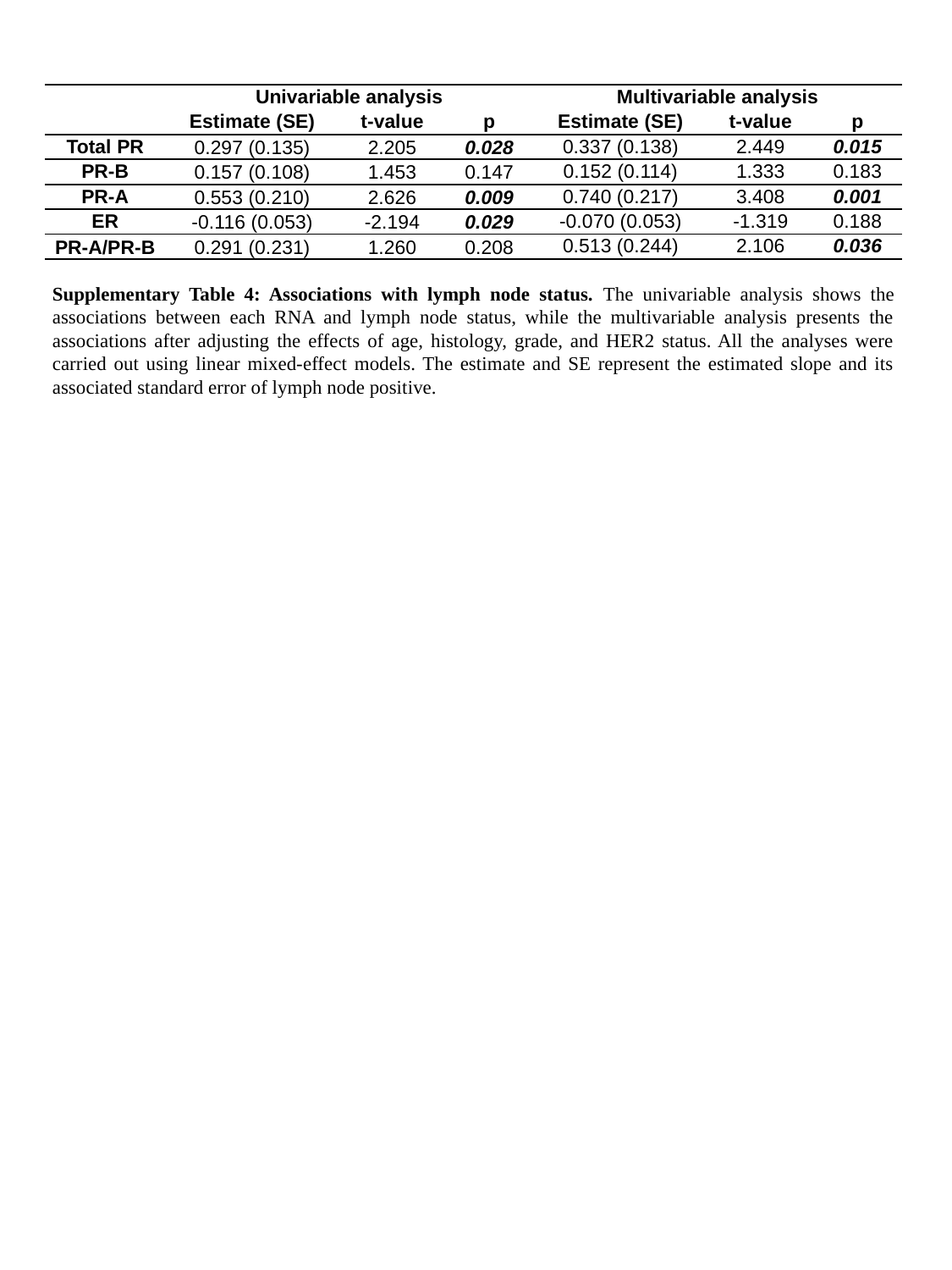

| | Univariable analysis | | | Multivariable analysis | | |
| --- | --- | --- | --- | --- | --- | --- |
| | Estimate (SE) | t-value | p | Estimate (SE) | t-value | p |
| Total PR | 0.297 (0.135) | 2.205 | 0.028 | 0.337 (0.138) | 2.449 | 0.015 |
| PR-B | 0.157 (0.108) | 1.453 | 0.147 | 0.152 (0.114) | 1.333 | 0.183 |
| PR-A | 0.553 (0.210) | 2.626 | 0.009 | 0.740 (0.217) | 3.408 | 0.001 |
| ER | -0.116 (0.053) | -2.194 | 0.029 | -0.070 (0.053) | -1.319 | 0.188 |
| PR-A/PR-B | 0.291 (0.231) | 1.260 | 0.208 | 0.513 (0.244) | 2.106 | 0.036 |
Supplementary Table 4: Associations with lymph node status. The univariable analysis shows the associations between each RNA and lymph node status, while the multivariable analysis presents the associations after adjusting the effects of age, histology, grade, and HER2 status. All the analyses were carried out using linear mixed-effect models. The estimate and SE represent the estimated slope and its associated standard error of lymph node positive.
