# Supplementary material for: Clinical association of progesterone receptor isoform A with breast cancer metastasis consistent with its unique mechanistic role in preclinical models
Source: BMC Cancer. 2020 Jun 3;20:512. doi: 10.1186/s12885-020-07002-0 (PMC7268268; doi:10.1186/s12885-020-07002-0)
Supplement: Supplementary file 2 — Additional file 2: Figure S1. The distribution of GAPDH Ct values (a) and the associations between GAPDH Ct value and each of lymph node (b), histologic type (c), and grade (d). In (c)-(d), the horizontal bars indicate a mean value and the p-value was calculated using repeated measures two-sample t-test and one-way ANOVA under linear mixed-effects models. [file 12885_2020_7002_MOESM2_ESM.pptx]

## Slide 1
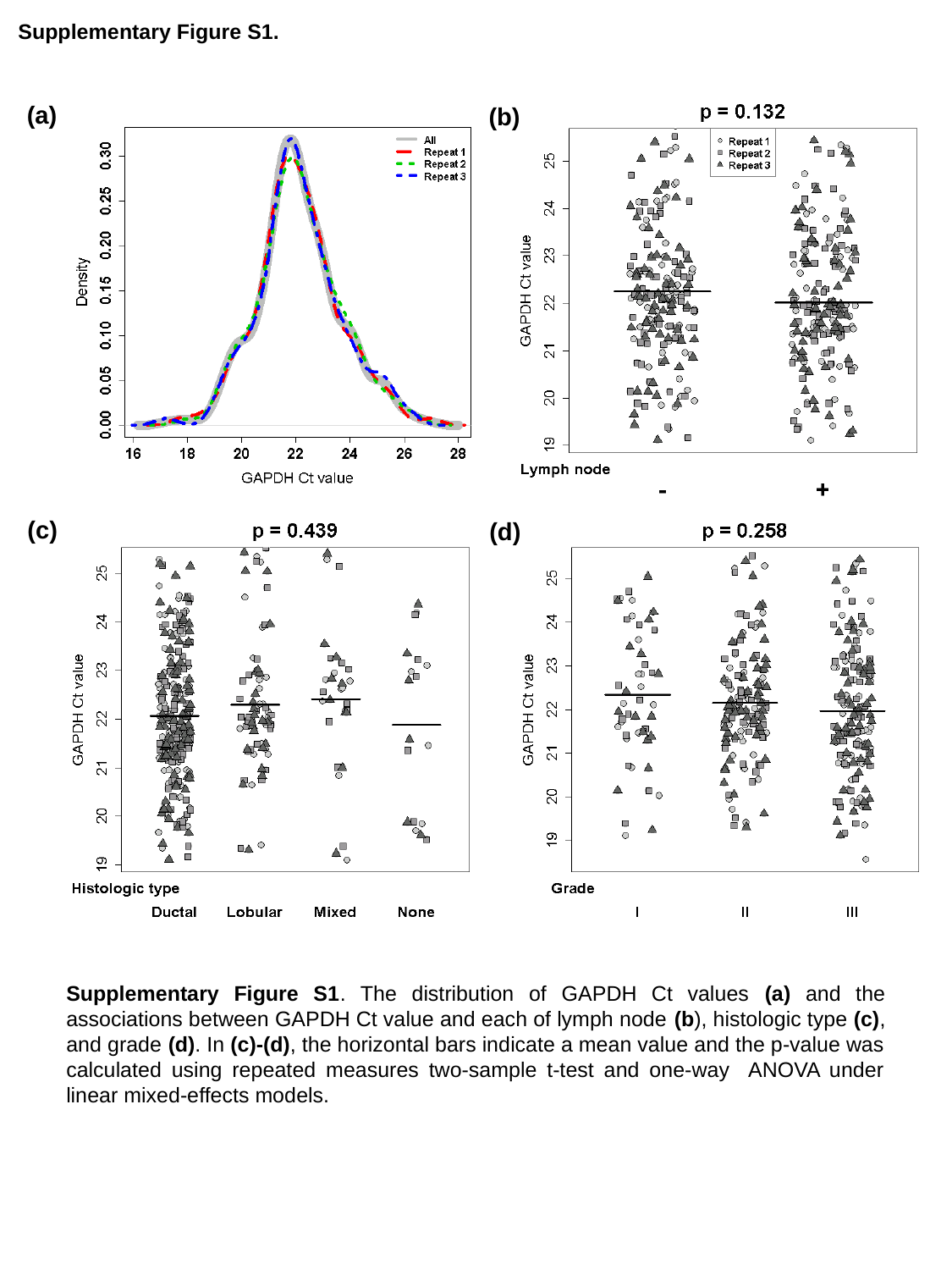

Supplementary Figure S1.
(a)
(b)
(c)
(d)
Supplementary Figure S1. The distribution of GAPDH Ct values (a) and the associations between GAPDH Ct value and each of lymph node (b), histologic type (c), and grade (d). In (c)-(d), the horizontal bars indicate a mean value and the p-value was calculated using repeated measures two-sample t-test and one-way ANOVA under linear mixed-effects models.
